# Amino Terminal Region of Dengue Virus NS4A Cytosolic Domain Binds to Highly Curved Liposomes

**DOI:** 10.3390/v7072812

**Published:** 2015-07-21

**Authors:** Yu-Fu Hung, Melanie Schwarten, Silke Hoffmann, Dieter Willbold, Ella H. Sklan, Bernd W. Koenig

**Affiliations:** 1 Institute of Complex Systems, Structural Biochemistry (ICS-6), Forschungszentrum Jülich, 52425 Jülich, Germany; E-Mails: y.hung@fz-juelich.de (Y.-F.H.); m.schwarten@fz-juelich.de (M.S.); si.hoffmann@fz-juelich.de (S.H.); d.willbold@fz-juelich.de (D.W.); 2 Institut für Physikalische Biologie, Heinrich-Heine-Universität Düsseldorf, Universitätsstraße 1, 40255 Düsseldorf, Germany; 3 Department Clinical Microbiology and Immunology, Sackler School of Medicine, Tel Aviv University, Tel Aviv 69978, Israel; E-Mail: sklan@post.tau.ac.il

**Keywords:** Dengue virus (DENV), non-structural protein 4A (NS4A), amphipathic helix, curvature sensing, peptide membrane interaction

## Abstract

Dengue virus (DENV) is an important human pathogen causing millions of disease cases and thousands of deaths worldwide. Non-structural protein 4A (NS4A) is a vital component of the viral replication complex (RC) and plays a major role in the formation of host cell membrane-derived structures that provide a scaffold for replication. The N-terminal cytoplasmic region of NS4A(1–48) is known to preferentially interact with highly curved membranes. Here, we provide experimental evidence for the stable binding of NS4A(1–48) to small liposomes using a liposome floatation assay and identify the lipid binding sequence by NMR spectroscopy. Mutations L6E;M10E were previously shown to inhibit DENV replication and to interfere with the binding of NS4A(1–48) to small liposomes. Our results provide new details on the interaction of the N-terminal region of NS4A with membranes and will prompt studies of the functional relevance of the curvature sensitive membrane anchor at the N-terminus of NS4A.

## 1. Introduction

Dengue virus (DENV), the causative agent of dengue fever, is a positive strand RNA, enveloped virus belonging to the *Flaviviridae* family. The viral RNA is translated into a single polyprotein which is processed by cellular and viral proteases into three structural proteins (capsid, premembrane, and envelope) and the seven non-structural (NS) proteins NS1, NS2A, NS2B, NS3, NS4A, NS4B, and NS5 [1]. The NS proteins are not found in the mature virion but are crucial for viral replication. Synthesis of viral RNA takes place in replication complexes (RCs) that contain essential NS proteins, viral RNA and host cell factors [2]. Upon DENV infection, a complex and continuous network of ER membrane-derived vesicular structures and convoluted membranes is formed. These structures contain the viral replication complexes and the sites of virion assembly [3,4,5].

NS4A is small integral membrane protein containing four predicted transmembrane segments (pTMSs) [1]. Although pTMS4, often referred to as the 2k fragment, is not part of the mature NS4A, it serves as a signal peptide for the ER localization of NS4B and is cleaved from the mature NS4A [6]. Experimental data verify that pTMS1 and pTMS3 span the membrane while pTMS2 is embedded in the luminal leaflet of the ER membrane and does not span it [1].

NS4A is crucial for the formation of the virus-induced membrane structures. Expression of NS4A lacking the 2k fragment alone is sufficient to induce membrane alterations that resemble the DENV-induced highly curved membranes that harbor the RCs [1]. Clearly, to induce these structures NS4A will have to closely interact with host membranes. However, the mechanism by which NS4A induces the curved morphology of these newly formed membranes is still unknown. Insertion of amphipathic helices into one leaflet of a membrane bilayer, as well as oligomerization of membrane proteins are among the mechanisms known to participate in the induction of membrane curvature [7]. Molecular dynamics (MD) simulations suggest that pTMS2 of NS4A could support membrane undulations upon stable association with the membrane [8]. Curved vesicular structures might also be induced via homooligomerization of NS4A [9,10]. While we have previously shown that the NS4A N-terminal cytoplasmic region is implicated in its oligomerization [10], a recent study demonstrated that pTMS1 is the major determinant in this process [9]. Introduction of two point mutations at the N-terminal of NS4A (L6E and M10E) reduced both the amphipathic character of this region and NS4A homooligomerization and abolished viral replication [10].

NS4A is an essential component of the viral RC [1]. Direct interaction of NS4A with the cytoskeletal protein vimentin was reported to be necessary for correct localization of the RC at the perinuclear site [11]. The vimentin binding site was found to be located at the N-terminal 50 residues of NS4A [11]. NS4A was also reported to bind NS4B, another component of the RC, via pTMS1 [9]. Mutational analysis suggests a functional relevance of this interaction for viral replication [9]. It was speculated that the interaction between NS4A and NS4B in concert with NS4A oligomerization and NS4B dimerization may play a role in the spatial and temporal regulation of distinct molecular complexes involved in the viral infection cycle [9].

Our previous circular dichroism (CD) data demonstrated that NS4A(1–48) interacts with highly curved small unilamellar liposomes under α-helix formation, while mutated NS4A(1–48, L6E;M10E) does not [12]. Surface plasmon resonance data indicated a seven fold-greater association of wild type NS4A(1–48) with immobilized liposomes compared to the mutant [12]. The structure of NS4A(1–48) in presence of membrane mimicking SDS micelles was characterized by NMR [12]. To further extend these results we used liposome flotation for direct proof of NS4A(1–48) binding to free liposomes. The exact location of lipid binding sites in the amino acid sequence of NS4A(1–48) was addressed by NMR spectroscopy. Our findings provide a basis for specific structure-function studies that will enhance our understanding of the role of NS4A and might provide future targets for anti-viral intervention.

## 2. Materials and Methods

### 2.1. Peptide Production

The peptide NS4A(1–48) corresponds to amino acid residues 1–48 from the *N*-terminal of NS4A of dengue virus serotype 2 (NCBI Protein database accession number: NP739588). A mutant peptide containing the mutations L6E and M10E was designated NS4A(1–48, L6E;M10E). The two NS4A peptides were recombinantly produced in *E. coli* BL21 cells and enzymatically cleaved from the affinity tag as described earlier [13]. Uniform isotope labeling with ^15^N or ^13^C, ^15^N was achieved by expression in M9 medium containing ^15^N ammonium chloride and ^13^C glucose (Eurisotop, Saarbrücken, Germany) as the sole source of nitrogen and carbon, respectively. Unlabeled peptides were expressed in LB media.

### 2.2. Fluorescence Labeling

Alexa Fluor 488 succinimidyl ester (NHS ester) was purchased from Life Technologies, Darmstadt, Germany. The dye was dissolved in anhydrous DMSO at a concentration of 3 mM immediately prior to the labeling reaction. For the reaction 300 μL from a 100 μM NS4A(1–48) or NS4A(1–48, L6E;M10E) stock in sample buffer (50 mM sodium phosphate, pH 6.8, 150 mM NaCl) were combined with 100 μL of 0.4 M NaHCO_3_ and the pH was adjusted to 8.3. This 400 μL peptide solution was supplemented with 100 μL of the Alexa Fluor 488 NHS ester in DMSO resulting in an approximately ten-fold excess of dye-over-peptide. The labeling reaction was wrapped in aluminum foil and incubated on a rocking platform shaker at 4 °C for 16 h. Labeled protein and free dye were separated on a Superdex 75 10/300 GL column (GE Healthcare, Freiburg, Germany) operated on an ÄKTApurifier system (GE Healthcare).

### 2.3. Liposome Preparation

The lipid 1-palmitoyl-2-oleoyl-*sn*-glycero-3-phosphocholine (POPC) in chloroform solution was purchased from Avanti Polar Lipids (Alabaster, AL, USA). Small unilamellar lipid vesicles (SUVs) were prepared from chloroform-free POPC dispersions (20 mg·mL^−1^) in sample buffer as described earlier [12]. SUVs were obtained by sequential extrusion through 50 nm (15 times) and 30 nm (15 times) Nuclepore polycarbonate membranes (GE Healthcare) with nominal pore diameter of either 50 or 30 nm, followed by sonication with a 3 mm microtip of a Branson 250 sonifier (15 cycles of sonication, 20 s each, interrupted by cooling for 2 min after each cycle). Sonicated SUVs were centrifuged for 10 min at 16,100× *g* and 10 °C in a refrigerated Eppendorf 5415 R tabletop centrifuge to remove any titanium abrasion of the microtip from the sample. The hydrodynamic radius of each liposome preparation was determined by dynamic light scattering (DLS) using a Dyna Pro instrument (Protein Solutions, Lakewood, NJ, USA) equipped with a 3 mm path length 45 μL quartz cell. Liposome solutions (20 mg of POPC per mL) were diluted 100-fold with buffer directly after extrusion or sonication and measured immediately. Data were analyzed with Dynamics V6 software distributed with the instrument. Experimental data were fitted to the model of Rayleigh spheres.

### 2.4. Liposome Floatation Assay

**Figure 1:**
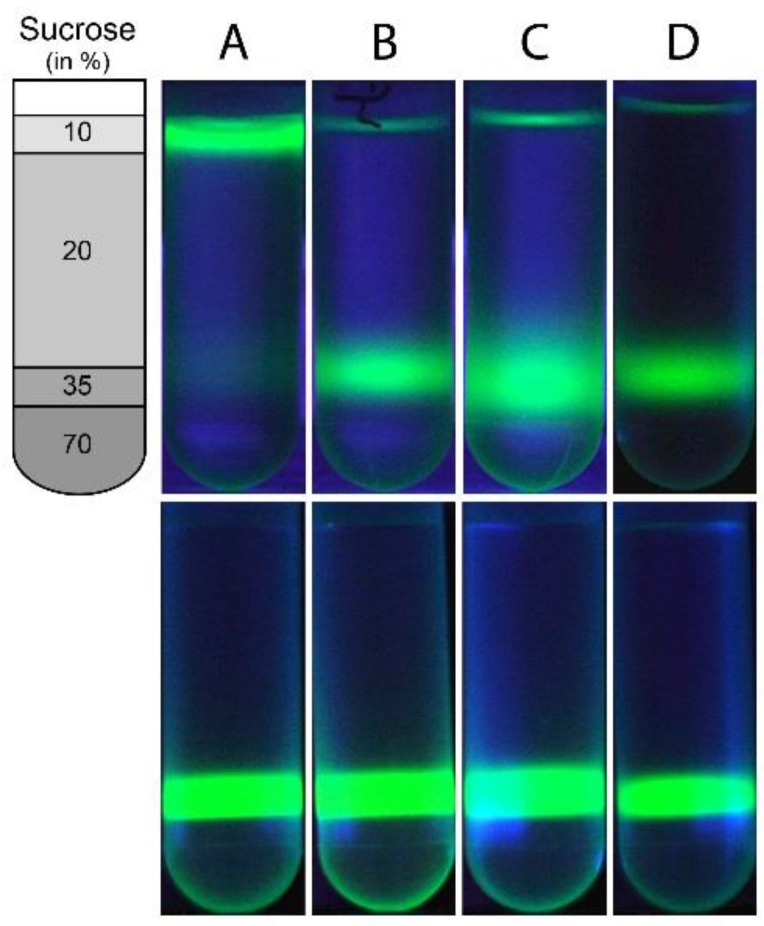
Liposome floatation assay of wild type and mutant NS4A(1–48). Alexa-488-labeled NS4A(1–48) (**A**); Alexa-488-labeled NS4A(1–48, L6E;M10E) (**B**); or free Alexa Fluor 488 dye (**C**) were mixed with sonicated POPC liposomes and loaded with the 35% (*w*/*v*) sucrose layer of a sucrose step gradient schematically shown on the left; Alexa-488-labeled NS4A(1–48) without liposomes was loaded with the 35% (*w*/*v*) sucrose layer in lane (**D**). Note: The narrow green line at the top of tubes (**B**), (**C**) and (**D**) results from reflection of fluorescence light at the air buffer interface rather than the presence of dye. Fluorescence images of the four tubes recorded prior to ultracentrifugation are shown in the lower row.

Equal volumes of 80 μM suspensions of Alexa Fluor 488-labeled peptides or free dye and sonicated POPC SUV (20 mg·mL^−1^) in sample buffer were combined and mixed at room temperature for 5 min. 100 μL of each of the three resulting samples were thoroughly mixed with 100 μL of a 70% (*w*/*v*) sucrose solution to obtain homogeneous solutions containing 20 μM of either Alexa Fluor 488-labeled peptide or free Alexa Fluor 488 dye, POPC liposomes (5 mg·mL^−1^) and 35% (*w*/*v*) sucrose in sample buffer. Sucrose solution (400 μL of a 70% (*w*/*v*) solution in sample buffer) was transferred to the bottom of a Polyallomer centrifuge tube (11 mm × 34 mm; Beckman Coulter) followed by a second layer formed by the 200 μL of 35% (*w*/*v*) sucrose solution containing one of the labeled NS4A peptides or the free dye and POPC liposomes. Finally, each sample was carefully overlaid with two cushions of decreasing sucrose concentration, *i.e.*, 1.2 mL of 20% (*w*/*v*) followed by 200 μL of 10% (*w*/*v*) sucrose in sample buffer (cf. scheme in Figure 1, left). Samples were centrifuged for 14 h at 259,000× *g* and 4 °C in a Beckman Coulter Optima Max-XP ultracentrifuge using a TLS 55 swinging bucket rotor. Fluorescence images were taken in front of a Mini Transilluminator (Bio-Rad, Munich, Germany) prior to and immediately after centrifugation.

### 2.5. Nuclear Magnetic Resonance (NMR) Spectroscopy

NMR experiments were conducted at 30 °C on Bruker Avance III HD NMR and Varian VNMRS instruments, equipped with cryogenic Z-axis pulse-field-gradient (PFG) triple resonance probes operating at proton frequencies of 700 and 900 MHz, respectively. Samples for resonance assignment contained 300 μM [*U*-^15^N, ^13^C]-labeled NS4A(1–48) in sample buffer (50 mM sodium phosphate, pH 6.8, 150 mM NaCl) as used for the liposome flotation experiments but supplemented with 10% (*v*/*v*) deuterium oxide and 0.03% (*w*/*v*) NaN_3_ (referred to as NMR buffer). Assignment of protein backbone resonances was accomplished using a combined set of heteronuclear multidimensional NMR experiments: 2D (^1^H–^15^N)-HSQC [14,15], 2D (^1^H–^13^C)-HSQC [16], 3D HNCA [17], 3D BT-HNCO [18], and 3D HNcaCO [19]. ^1^H and ^13^C chemical shifts were referenced directly to internal 4,4-dimethyl-4-silapentane-1-sulfonic acid (DSS) at 0 ppm and ^15^N chemical shifts were referenced indirectly to DSS using the absolute ratio of the ^15^N and ^1^H zero point frequencies [20]. NMR data were processed using NMRPipe, v.8.1 [21] and evaluated with CcpNmr v.2.4 [22].

The interaction of NS4A(1–48) and NS4A(1–48, L6E;M10E) with sonicated POPC liposomes was studied based on a series of 2D (^1^H–^15^N) Heteronuclear Single Quantum Coherence (HSQC) spectra recorded with 40 μM peptide in NMR buffer but with different amounts of liposomes (0; 2.5; 5, or 10 mg of POPC per mL). Spectra were acquired with 150 complex data points in the ^15^N time domain, up to 128 scans per *t*1 increment and a recycle delay of 1.5 s. Data were processed and analyzed using NMRPipe.

## 3. Results

### 3.1. Wild Type NS4A(1–48) Binds to Highly Curved POPC SUVs

Binding of fluorescently labeled proteins to liposomes can be visualized using a simple floatation assay [23]. POPC bilayers have a mass density very close to 1 g·cm^−3^ [24] at room temperature. Thus, POPC liposomes will migrate to the top layer containing the lowest sucrose concentration in a step gradient of decreasing sucrose concentrations upon centrifugation. In contrast, a small protein of ~5 kDa molecular weight is expected to have a mass density well above 1.41 g·cm^−3^ [25] and thus will accumulate in a sucrose rich layer of high mass density. Alexa-488-labeled NS4A(1–48) apparently binds to small sonicated POPC liposomes and migrates with the liposomes to the top of the gradient (Figure 1A). In contrast, the mutated peptide NS4A(1–48, L6E;M10E) remains in the high density layer with 35% (*w*/*v*) sucrose even after 14 h of centrifugation (Figure 1B), indicating that this peptide does not bind to liposomes. As a negative control, the Alexa-488 free dye was loaded to the 35% (*w*/*v*) sucrose layer with POPC liposomes. The free dye remained in the high-density region after centrifugation (Figure 1C) indicating a lack of association with the POPC SUVs. The fluorescent band of the free dye has a larger vertical extension than that of the labeled NS4A(1–48, L6E;M10E) after 14 h of centrifugation (Figure 1B,C). This probably reflects the larger diffusion coefficient of the low molecular weight dye molecule (643.4 g·mol^−1^). In a second control experiment Alexa-488-labeled NS4A(1-48) was loaded to the 35% (*w*/*v*) sucrose layer without adding POPC liposomes. As expected, the peptide did not significantly migrate during the 14 h of centrifugation and remained almost completely in the 35% (*w*/*v*) sucrose layer (Figure 1D). The floatation results confirm the binding of the wild type peptide to small liposomes.

### 3.2. NMR Identifies Regions of NS4A(1–48) Associated with POPC Liposomes

NMR spectroscopy was used to identify the regions of NS4A(1–48) associated with POPC liposomes. Almost complete assignment of the expected ^1^HN, ^15^N, ^13^Cα and ^13^C’ backbone resonances was accomplished for both NS4A(1–48) and NS4A(1–48, L6E;M10E) in NMR buffer. No assignments were obtained for S1 and N42. In addition to P14 there are four residues which only lack amide (^1^HN, ^15^N) assignments. Assignments have been deposited at the Biological Magnetic Resonance Data Bank (BMRB) under accession number 25586 for NS4A(1–48) and accession number 25676 for NS4A(1–48, L6E;M10E).

**Figure 2:**
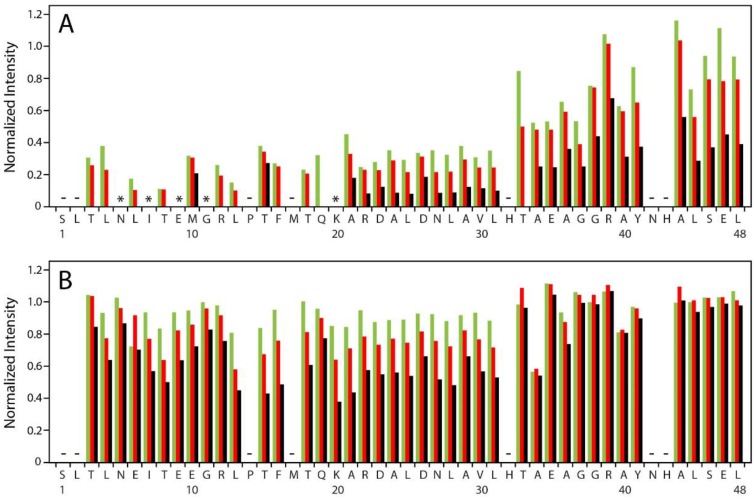
Intensity of backbone amide cross peaks in HSQC spectra of NS4A(1–48) (**A**) and NS4A(1–48, L6E;M10E) (**B**) recorded at various lipid concentrations. Peak intensities measured at 2.5 (green), 5 (red) and 10 mg·mL^−1^ POPC (black) in the sample were normalized to the intensity of the same signal observed in lipid-free buffer and are shown as a function of the amino acid sequence of the studied peptide. Cross peaks not observed in the lipid-free sample are indicated by minus signs. Cross peaks that are present in buffer but completely disappear after addition of 2.5 mg·mL^−1^ POPC are indicated by asterisk.

Amide ^1^HN, ^15^N cross peaks in HSQC spectra of NS4A(1–48) were used to monitor peptide interaction with sonicated POPC liposomes in an amino acid residue resolved manner. The hydrodynamic radius of the POPC liposomes was ~26 nm based on DLS measurements. Backbone cross peaks for most of the 48 amino acid residues of NS4A(1–48) were identified in buffer without liposomes except for S1, L2, P14, M17, H32, N42 and H43. The observed cross peaks characterize the free peptide conformation. Addition of increasing amounts of sonicated POPC liposomes at constant peptide concentration caused gradual peak intensity reductions in a peptide region specific manner (Figure 2A). Interestingly, peak positions did not significantly change, except for R12, which showed a small shift. Such a behavior is typical for slow or intermediate exchange of the peptide between the free and liposome-bound state. Strongest peak intensity reduction is observed in the N-terminal region extending up to K20 (Figure 2A). Some peaks in this region completely disappear already at 2.5 mg·mL^−1^ POPC (N5, I7, E9, G11, K20) while the others are reduced to less than 40%. At the highest liposome concentration studied (10 mg·mL^−1^) only two cross peaks from this peptide region remain visible and show low intensity. It is likely, that a number of amino acid residues of the N-terminal region bind directly to the liposome. Binding will change the chemical environment and strongly reduce the rotational correlation time of the amino acid residues in direct contact with lipids. NMR signals of bound residues are likely broadened beyond detection. Exchange dynamics may differ somewhat among the amino acid residues in this region, explaining the variable intensity reduction of the free state cross peaks.

Cross peaks of the central region from A21 through L31 of NS4A(1–48) show rather uniform intensity reduction upon gradual liposome addition (Figure 2A). All free state peaks remain visible and retain about 10% of their original intensity even in the presence of 10 mg·mL^−1^ POPC. Different scenarios might contribute to the reduction of the free state peak intensities. Peptides that are anchored with their N-terminal region in the liposome might retain their free state conformation in the central domain, albeit with a reduced overall rotational correlation time and thus lower peak intensities. In addition, amino acid residues of the central region of some NS4A peptides might bind directly to the liposome leading to the disappearance of the corresponding NMR signals. The uniform peak intensity reduction pattern in the central region may suggest a concerted binding of this amino acid stretch, e.g., as one secondary structure element, to the liposome.

Finally, amino acid residues in the C-terminal region of NS4A(1–48) from T33 to L48 show the smallest reduction in the free state peak intensities upon liposome addition (Figure 2A). Perhaps, these small intensity reductions might be entirely caused by anchoring of the peptides via amino acid residues in the N-terminal and perhaps the central regions.

### 3.3. NMR Data Suggest Diminished Interaction of NS4A(1–48, L6E;M10E) with POPC Liposomes

Inspection of HSQC spectra of mutant NS4A(1–48, L6E;M10E) in buffer and with increasing amounts of sonicated POPC liposomes revealed no changes in cross peak positions and only a minor influence of lipid addition on cross peak intensities of backbone amide signals. All cross peaks observed in buffer remain visible in presence of liposomes and retain at least 40% of their original intensity upon addition of 10 mg·mL^−1^ of POPC liposomes (Figure 2B). Almost no peak reduction is observed for the C-terminal region of NS4A(1–48, L6E;M10E). Peak intensity reductions in the N-terminal and central regions are quite moderate, in particular at low liposome concentration (2.5 and 5 mg·mL^−1^ POPC). Even at 10 mg·mL^−1^ POPC liposomes cross peak intensities remain between 40% and 80% (N-terminal region) or between 40% and 60% (central region) with respect to intensities measured in buffer. Apparently some interaction of the mutant peptide with liposomes is retained at the highest lipid concentration studied. Amino acid residues in the central and perhaps in the N-terminal part of the mutant peptide are likely to make transient contact with the liposome. However, the observed peak intensity reductions are much weaker in case of NS4A(1–48, L6E;M10E) than for wild type.

## 4. Discussion

The L6E and M10E mutations that disrupt the amphipathic character of the NS4A *N*-terminal abolish viral replication, indicating that the 48 N-terminal residues of NS4A play a crucial role in replication. Furthermore, these mutations had a similar effect when inserted as single mutations [10]. We have previously shown that NS4A(1–48) interacts preferentially with highly curved liposomes. CD spectroscopy demonstrated that these two point mutations severely compromise this interaction [12]. The interaction of wild type NS4A(1–48) with highly curved membranes has been demonstrated for three different lipid compositions, *i.e.*, pure POPC, a POPC/DOPS mixture at a molar ratio of 4:1, and a blend of synthetic lipids resembling the composition of membranes in the ER (ER lipid mix), but no dependence on lipid composition was detected [12]. Here, we confirm these results using a liposome floatation assay and NMR. Initial floatation experiments were conducted with pure POPC and with the ER lipid mix. Again, no influence of lipid composition on NS4A(1–48) binding was observed. The detailed analysis presented in the current manuscript was conducted with single component POPC SUVs. Liposome floatation experiments (Figure 1) clearly show that wild type NS4A(1–48) binds to highly curved POPC liposomes, this interaction was not observed with the mutant peptide. The interaction between NS4A(1–48) and liposomes was further characterized using NMR. These experiments indicate that the main lipid binding sites of the peptide are located at the N-terminal 20 amino acid residues of NS4A(1–48). Residues A21 through L31 may also be involved in liposome binding while the remaining C-terminal residues are only weakly affected by liposome binding and do not seem to play a direct role in this process. The backbone resonance assignment of NS4A(1–48) and NS4A(1–48, L6E;M10E) reported in this manuscript contains information on the secondary structure of the two peptides in lipid-free buffer. Analysis with the TALOS-N software [26] clearly shows the lack of secondary structure for both peptides in buffer. The NMR data on NS4A(1–48) recorded in presence of liposomes do not allow any straightforward conclusion on the structure of the liposome-bound peptide. However, interaction of NS4A(1–48) with sodium dodecyl sulfate (SDS) micelles induces the formation of two amphipathic helices (AH) encompassing residues N5 to E9 (AH1) and T15 to L31 (AH2) of NS4A(1–48) [12]. CD spectra of NS4A(1–48) recorded in presence of either SDS micelles or small POPC liposomes are very similar [12]. Therefore, it is conceivable that the two amphipathic helices AH1 and AH2 are also formed in the liposome-bound peptide. The *N*-terminal region of NS4A(1–48) that forms AH1, an interhelical linker and the N-terminal half of AH2 in SDS micelles seem to be crucial for peptide binding to liposomes. Interestingly, this region also contains the two mutations L6E and M10E, which abolished liposome binding in the floatation experiment (Figure 1). Moreover, mutagenesis of other residues in this region including P14A [10], R12A and K20A [9] was also shown to reduce or abolish DENV replication.

The central part of NS4A(1–48) encompassing residues A21 to L31 shows less pronounced NMR signal intensity reductions upon titration with POPC liposomes than its N-terminal 20 amino acid residues (Figure 2A). A comparatively weak NMR signal intensity reduction is observed for both the N-terminal and central regions of NS4A(1–48, L6E;M10E) (Figure 2B) indicating some residual interaction with liposomes. However, the liposome floatation assay clearly shows that this interaction of the mutant peptide with POPC liposomes is too weak for stable anchoring of the peptide at the membrane. The amino acid sequence of A21 through L31 is identical in both peptides. We conclude that this amino acid stretch A21 to L31 is not sufficient for stable membrane anchoring of NS4A(1–48).

DENV NS4A apparently contains two separate membrane anchors. The membrane spanning helices pTMS1 and pTMS3 stably integrate the protein into the membrane. The N-terminal region of the cytosolic domain specifically binds to the convex surface of highly curved membranes [12] and may serve as a second membrane anchor. Therefore, one hypothesis might be that NS4A can bridge two adjacent membranes or connect separate patches of the same membrane that come into close proximity due to membrane convolution. We speculate that membrane bridging by NS4A might play a crucial role in stabilizing the complex morphology of DENV-induced ER-derived membrane structures, which include stacks of convoluted membranes (CM), double membrane vesicles and tubes [5]. The vesicles were described as invaginations of the ER, which are connected to the cytosol via pore-like openings [5]. NS4A may play different roles in the reorganization of these ER-derived membranes. Asymmetric insertion of pTMS2 into the luminal leaflet of the inner membrane of the vesicles as well as oligomerization of NS4A may induce concave membrane curvature required for vesicle formation [7]. Binding of the N-terminal region of NS4A to the saddle-shaped neck region connecting the vesicle and the pore may further stabilize the vesicular structures.

All positive-strand RNA viruses form their replication complexes on modified host membranes. However, the source of the membranes and the nature of the modifications vary (for review see [2]). In general, the role of these modifications is twofold; to provide a scaffold concentrating and correctly positioning the viral and host factors for efficient viral replication and to protect the replicating virus from detection by the host immune system. The mechanisms driving the formation of these structures are still incompletely understood. Convoluted membranes are a form of membrane modification induced by several positive-strand RNA viruses including, in addition to DENV, severe acute respiratory syndrome coronavirus (SARS-CoV) and Kunjin virus (KUNV) [5,27,28], for example. In KUNV convoluted membranes are thought to be the site of polyprotein processing [27]. While in DENV the role of these structures is still unclear, they are thought to be a depot for factors required for replication [5].

In summary, the liposome floatation data provide direct proof for specific binding of NS4A(1–48) to highly curved free liposomes. The main lipid binding sites in NS4A(1–48) are located within the N-terminal 20 amino acid residues. The exact role of this specific interaction in the viral life cycle is still under investigation. Nevertheless, this important structural information may assist in further understanding of the role of NS4A and the mechanism by which it induces the membrane alterations underlying the viral RC formation.
